# The relationship among safety leadership, risk perception, safety culture, and safety performance: Military volunteer soldiers as a case study

**DOI:** 10.3389/fpsyg.2023.1000331

**Published:** 2023-02-23

**Authors:** Siao-Yun Wei, Yen-Ku Kuo

**Affiliations:** ^1^Department of Banking and Finance, Commercial College, Chinese Culture University, Taipei, Taiwan; ^2^Bachelor Program of Leisure Management, Commercial College, Chinese Culture University, Taipei, Taiwan

**Keywords:** safety leadership, risk perception, safety culture, safety performance, military volunteer

## Abstract

Safety is fundamental to any organization; if not based on safety, organizational decision-making and management would be meaningless. For a country, soldiers are responsible for national security; they serve as a barrier that defends a country against external invasive forces, thus assuming great missions and responsibilities on their shoulders. To ensure soldiers fulfill their duties of protecting the country and the people, they should gain clear risk perception, which should be instilled into them during their daily combat readiness training. Only when their performances meet safety criteria can they become a strong fighting force. This study recruited military volunteer soldiers as its research participants and employed convenience sampling to distribute questionnaires. In total, this research collected 725 valid copies, of which the data were used to explore the relationship among safety leadership, risk perception, safety culture, and safety performance. To achieve this goal, this study proposed some research hypotheses based on literature review. The hypotheses were all verified *via* latent variable modeling and multiple hierarchical regression analysis after the reliability and validity of each construct had been tested *via* confirmatory factor analysis. The research results showed that the more deeply military volunteer soldiers sense safety leadership, the clearer their risk perception will be and the more helpful it would be in achieving safety performance. It is worth mentioning that risk perception can serve as a mediator while safety culture can mediate the relationship between safety leadership and safety performance. Lastly, the research proposes suggestions in the section of conclusions, which provides reference to the combat readiness training and daily tasks of soldiers.

## 1. Introduction

Soldiers, a force that ensures the peace and stability of a society, play an important role in safeguarding national security. Along with the change and reform of national defense policies, a country is in greater need of a smaller army with quality soldiers, striving for precise and safe implementation of military tasks. In this context, soldiers are expected to know more about safety codes of practice (Cherkasov et al., 2016). Soldiers serve as a fundamental force that protects a country and guarantees military organizational security. Thus, the manpower of military organizations is often complicated and elaborate, which necessitates effective leadership to achieve effective command (Dupont, 2017). In this respect, safety leadership is vital to military organizations; only with military officials’ effective safety leadership can military subordinates gain clear and accurate risk perceptions, which facilitates accomplishing safety performance when they undertake tasks. In addition, overall organizational culture embodies the values behind each event; in an organization that values safety culture, soldiers will find it natural and normal to conform to safety standards and specifications when carrying out their tasks, big or small.

Military organizations often have sophisticated equipment, and even their own unique production systems and plants. To effectively operate these systems, soldiers need to pay much attention to safety standards and operating procedures in addition to essential knowledge and skills, which will help them raise combat readiness and increase combat power (Santtila et al., 2019). During daily military training, soldiers often need to utilize weapons or operate military systems. If they are not careful, they will lead to accidents that cause damage and life loss. From this perspective, safety is vitally important to military organizations. The main purposes of safety behaviors and safety management are to reduce accidents and to complete tasks more safely (Merritt, 2020). The damages incurred by daily tasks or combat readiness drills are often avoidable and can be prevented beforehand, for they are often caused by carelessness. Therefore, it is necessary to foster safety culture within an organization because safety culture of an organization will instill safety perception into its members in an imperceptible way, thus ensuring the safety of each member. Due to its strategic location, Taiwan has always played a balancing act between the United States and China, two large countries (Wang, 2018). Political and historical factors have led to the need for Taiwan to continuously resist the threat of Chinese force, and therefore, Taiwan needs to maintain good tactical readiness and warfare. Such issues arise in every aspect of daily training activities, so a culture of security and a security attitude is undoubtedly important to Taiwan’s military (Wu et al., 2022).

Members of Taiwan’s military have both volunteer and volunteer status. Generally, adult men in Taiwan are required to undergo military training and serve for approximately 1 year (Golby et al., 2018). During the time that adult Taiwanese men go through military training, they are given a lot of knowledge and skills in warfare and tactics to ensure that each member has the basic ability to handle war. Even after completing their military training, Taiwanese men may still receive occasional call-ups and may have to return to the military at any time for additional training in various tactics (Green and Talmadge, 2022).

Consequently, military officials should have a good understanding of the influence of safety leadership, which would help them deal with complicated tasks and attain a great safety performance. This way, an army is able to complete tasks, guarantee combat power, and give full play the qualities and talents of its soldiers (Romaní-Romaní et al., 2016). Whether for daily work or for combat readiness training, the safety literacy of a soldier should be valued and communicated by all military organizations. Only when leaders set an example in exhibiting safety behaviors or clearly explain standard operating procedures when giving orders will soldiers manage to ensure safety when performing tasks (Karhina et al., 2017). It is of great importance for soldiers to have risk perception for each event because in most cases, they need to perform tasks on their own rather than in a group; only with risk perception will soldiers raise their awareness of prevention when carrying out daily tasks and receiving training. Due to the technological advancement and social transformation, an army is not built up merely based on its ability to fight a battle (Hanif et al., 2019); soldiers engage in disaster relief tasks in many countries. In other words, military organizations should have clear safety perception in order to achieve good safety performance in all of their tasks. In this regard, it is of significance to explore the relationship among safety leadership, risk perception, safety culture, and safety performance. Consequently, this study aims to understand the safety awareness of soldiers by proposing hypotheses based on literature review and employing statistical methods to verify them, based on which relevant suggestions were put forward.

## 2. Literature review

### 2.1. Safety leadership

Leaders are like a guiding light on the sea; leaders are expected to point subordinate’s directions when they are faced with problems rising from quickly changing environment and aim to create a future. Leadership means much more in a fast-changing era. The trait theory of leadership, the behavioral theory of leadership, and the contingency theory of leadership have all revealed that leadership of an organization provides a glimpse of how successful it is. This also applies to safety leadership. The overall performance is closely related to the interaction between superiors and subordinates and the coordination between control and influence brought by a situation (Pilbeam et al., 2016). Safety leadership refers to the safety vision and policies developed by leaders to influence organizational members through setting examples, thus achieving safety goals in a concerted manner (Wu et al., 2016). Safety leadership is about an individual leading and influencing others or groups to realize safety goals while completing organizational tasks; the leadership and influence appears throughout the whole process. As for safety leaders, they are those striving to exert an impact and exercise safety leadership (Sheehan et al., 2016). Jiang and Probst (2016) defined safety leaders as those who promote safety measures to realize safety vision with human care and set examples. Mullen et al. (2017) believed that safety leaders should outline their own safety vision and set it as a model, empowering their members to participate in safety planning and decision-making to win the members’ trust, which cannot be replaced by safety managers (Fernández-Muñiz et al., 2017). Wu et al. (2017) held that safety leadership is a kind of leadership that shows the importance that leaders attach to safety-related issues, motivating organizational members to have a positive impact on work safety. Furthermore, Oah et al. (2018) pointed out that safety leadership development can improve safety culture, thus enhancing safety performance. Safety leadership is indispensable to an organization if it means to cultivate safety culture and achieve excellent safety performance. Donovan et al. (2016) regarded effective leadership of managers as part of safety culture because safety leadership determines the viewpoints of organizational members on safety issues while Grill et al. (2017) believed that safety leadership is part of organizational leadership. Safety leaders must be the actors of transformation; they are responsible for keeping organizational members alert to ever-changing risk factors and managing risk control. Safety managers should know when to act as safety leaders, demanding their subordinates to know clearly about their job duties rather than holding the subordinates responsible for potential consequences. To become a safety leader, one should change his/her other-oriented motivation into self-oriented one. A potent safety leader should be able to exercise five sources of power to motivate and influence employees, namely coercive, reward, legitimate, expert, referent power in order to achieve safety goals (Kim and Gausdal, 2017). Zhang et al. (2018) defined safety leadership as leaders exercising their influence within an organization to achieve safety objectives throughout the interaction between leaders and employees. Stiles et al. (2018) claimed that safety leadership enables team members to work harder and more efficiently, advocating a responsibility system for safety performance. Therefore, safety leadership is the origin of ensuring the safety of an organization, and it is also the management element that can influence the members of an organization to understand the importance of safety (Kerin, 2022). Taiwan’s military is very concerned about the importance of safety to prevent the threat of force due to political sensitivity, so military leaders and soldiers need to know the importance of good and adequate safety leadership.

### 2.2. Risk perception

Risk perception is a process in which people make “subjective” judgments about an event due to the influence of daily life (Kapuściński and Richards, 2016). Operators are not able to predict the future change of the elements that constitute the environment due to the uncertainty of external information or they do not know well the relationship between the elements, thus perceiving risks (Hamid, 2020). Risk perception factors can be divided into five types, including physical environment, loss and compensation, individual social-economic attributes, social trust, and mentalities (Perpiña et al., 2019).

Risk perception is a judgment about negative results based on the conversion of probability to symbols or signs while the judgment is influenced by individual attributes, previous experience, information processing abilities, the severity of an event, willingness, and control abilities (Leder et al., 2019). Alexandrou and Chen (2019) believed that individuals mainly rely on their instinct to make judgments about risks when rating various risky matters, which is called risk perception. Oh et al. (2020) employed the following indicators to evaluate employees’ safety and measure emergencies: physical condition, attitude toward safety, and prevention of accidents at work, and found that risk perception is positively correlated to risky behaviors. Macias (2016) proposed that risk perception refers to the risks that decision-makers sense when they evaluate certain situations, which includes the way decision-makers describe the situation, risk control, probability estimation, and the confidence in estimation. Bodoque et al. (2016) believed that risk perception refers to the situation that people make evaluations on subjective quantitative assessments rather than on scientific measurements, and engage in various activities with their perceived results when assessing the risks, they may encounter in daily life. Wang et al. (2016) defined risk perception as safety warnings in a broad sense, in other words, one’s full understanding of possible hazards and potential consequences or a situation that can lead to potential harm. Ferrer et al. (2016) claimed that risk perception is an impression about or instinct for health risks and the judgment about risk intensity. Bronfman et al. (2016) explained people’s belief, attitude, judgment, feelings, and adventure about risks in social contexts and under cultural values. Sullivan-Wiley and Gianotti (2017) believed that risk perception refers to the response of individuals to external stimuli, which involves two processes, both physical and mental. Cui et al. (2016) believed that operators perceive risks when they cannot predict the future changes in the elements that constitute the environment due to the uncertainties of external information or when they do not know precisely the relationship between the elements. Zhu et al. (2016) pointed out that risk perception is the process in which people make judgments about events subjectively under the influence of daily life. As a consequence, risk perception is a judgment about negative results based on the conversion of probability to symbols or signs, which is influenced by individual attributes, previous experience, information processing abilities, the severity of an event, willingness, and control abilities. Therefore, risk perception should be a premonition that all members of an organization need to be aware of any impending threats. Risk prevention requires a lot of expertise and experience to gain an inspiration to prevent risks at critical times (Znajmiecka-Sikora and Sałagacka, 2022). Taiwan’s military officials and soldiers should cultivate risk perception at every moment because there is always a chance of a threat from war, so risk perception is a very important part.

### 2.3. Safety culture

Safety culture of an enterprise expands its corporate culture to workplace safety issues; it is related to the culture of corporate safety, which is reflected in organizational members’ safety attitudes, behaviors, beliefs, norms, and practice, which facilitates risk assessment, safety audit, training, and strategy management of enterprises. Viewed from individuals’ perspective, safety culture can improve the behavioral safety, safety knowledge, and safety motivation of workers, and lower the rate of personal injury, thus cultivating their safety awareness and safety behaviors (Berry et al., 2020). The development of a cultural system influences and shapes the safety values, beliefs, norms, and perceptions of organizational members (Danielsson et al., 2019), which enables them to be consistent in their awareness and perception in this respect. Thus, the members will hold homogenous safety beliefs and take similar safety actions and actively acquire relevant knowledge to exhibit their safety attitude and safety behavior. Organizational culture is a mixture of shared values, attitudes, and behaviors, which gives an organization a unique characteristic; in brief, it is a way that a group do things. In addition, Stemn et al. (2019a,b)believed that safety culture is shared ideas about the risks, accidents, and damages incurred by safety problems that all organizational members face. In this regard, Ross et al. (2019) deemed safety culture as the norms, beliefs, roles, attitudes of an organization, and the practice that it follows to lower the possibility of exposing its members to a dangerous work environment. Lee et al. (2019) believed that safety culture reflects shared attitudes, beliefs, perceptions, and values that employees hold in terms of safety. De Boeck et al. (2019) believed that safety culture is about common and basic settings of groups when they solve the problems that require external adaptation and internal integration, for the settings work well and are deemed effective. That is why they are imparted to new members to help them find right ways to solve problems. Schwartz et al. (2019) believed that safety culture is a subculture of an organization, which can be referred to trust, values, and specific safety and health. Similarly, Leaver and Reader (2019) believed that safety culture is a subsystem of organizational culture, which affects members’ attitude and thinking behind certain behaviors and plays a role in their safety performance. Moreover, Le Coze (2019) held that safety culture is about responsibility alternation and comprehensive performance, so it is necessary to construct the beliefs about danger and safety. In this respect, Tear et al. (2020) believed that safety culture is a key element that affects the cultivation of safety climate. Aboneh et al. (2020) defined safety culture as follows: for group members, in any group and at any level, public safety is a constantly valued priority, which provides reference to individuals and groups when they need to respond to safety and express safety concerns with actions. Wang and Wu (2019) held that the establishment of safety culture can prevent an organization from making mistakes, believing that the safety culture of an enterprise sets clear and concise procedures and provides training and vigilance in terms of the factors affecting safety and effective communication, which has a clear and stable organizational structure. Gao et al. (2019) believed that safety culture is common and shared values, attitudes, behavioral patterns, and rules that individuals and groups have about occupational safety in an enterprise while safety culture shapes its safety characteristics and safety climate. Teleş and Kaya (2019) believed that safety culture is the core safety values of all members within an organization, their common sense of safety perception and safety beliefs as well as positive attitudes toward safety. Safety culture is embodied in the explicit behavior of individuals that reflects their viewpoints, behaviors, thinking, actions, and propositions, which also includes the positivity that organizational members display to safety and prevention measures, safety responsibility that they assume, and their safety commitment. Cooper et al. (2019) claimed that people, behavior, and environment are fundamental elements of a safety culture; good management and leadership enable subordinates to sense the style of their superiors’ safety leadership, for which subordinates will reflect the safety values in their daily work. Timmermans et al. (2019) held that safety culture is a composite of shared attitudes, features and beliefs that organizational members are willing to uphold during the production in an organization to guarantee the safety of them and the organization and avoid potential damages; it is also noble values that they exhibit both behaviorally and mentally. Teperi et al. (2019) believed that safety culture is an extension of corporate culture, including the commitment of the management to safety, the improvement of safety and health equipment within an organization, and employees’ feelings about whether it implements organizational safety management. Kalteh et al. (2019) stated that safety culture is organizational members’ safety awareness, acquisition of safety knowledge, commitment to workplace safety, observation of safety standards and procedures, and their ability to respond to emergencies in a rapid and effective manner. Corrigan et al. (2019) believed that safety culture is a product of organizational members’ shared safety values, beliefs, attitudes, perceptions, and behavioral patterns, which determine an organization’s safety management style and safety performance. Safety culture allows an organization to think in terms of the core values of safety in everything it does, ensuring that every decision and action is aligned with the axis of safety (Byrnes et al., 2022). Taiwan’s military officers and soldiers need to be proactive and preventive in building a safety culture together, maintaining it in every detail of their activities in order to prevent the threat of force from attacking regional security.

### 2.4. Safety performance

Occupational health and safety management based on laws and rules has been transformed to the one with systematic and continuous improvement as its goal. Systematic occupational health and safety management aims to lower safety risks, prevent occupational disasters, and improve safety performance and management continuously. An organization can find out whether invested resources produce expected effects *via* performance evaluation so that the management can discern the advantages and disadvantages of the implementation of the workplace safety plan. An organization without occupational disasters does not necessarily have a good safety performance. In this respect, Stemn et al. (2019a,b) believed that early safety assessment of a company, a department, and its equipment *via* accident frequency and severity cannot precisely reveal whether its system is effective and under control and whether its diagnosis is correct. Performance evaluation is an essential task that assesses whether safety management measures are well implemented. The management’s safety monitoring and implementation as well as safety performance measurement show their efforts and determination to improve safety performance, which is an action that positively cultivates safety culture (Farid et al., 2019). Haas (2020) proposed that safety performance is an overall perception of safety-related issues while Singh and Verma (2019) defined safety performance as a shared perception of employees for their work environment. Similarly, Kaspers et al. (2019) held that safety performance is an overall perception of employees for their work environment, which affects their safety behaviors. Ghahramani and Salminen (2019) believed that safety performance is an objective measurement of organizational health and safety issues. Jana et al. (2019) pointed out that safety performance is a special organizational climate, revealing the value of workplace safety for individuals. Netjasov et al. (2019) put forward that safety performance is an overall perception of organizational members for safety. In addition, Kalteh et al. (2019) further pointed out that the biggest problem of safety lies in performance evaluation. Lu et al. (2019) found that safety training and safety management are optimal predictive factors that forecast safety performance based on their analysis of work groups. Sultana et al. (2019) believed that safety performance is a prediction of the probability of accident occurrence and safety. Chu et al. (2019) believed that safety performance evaluation facilitates the confirmation of advantages and disadvantages of a safety system, which helps effectively find out the cognitive gap between the management and employees and provides reference for safety improvement, thus gaining organizational competitive advantages. Wang et al. (2019) pointed out that safety performance is an overall performance that organizational members deliver regarding safety at work. Stemn et al. (2019a,b) defined safety performance as an overall evaluation of organizational safety, which can reveal the strength and weakness of a safety management system that serves as a basis for organizational improvement in terms of safety culture and competitiveness. Xu et al. (2019) mentioned in their research that in order to find out the safety performance of an organization’s operation, it is necessary to explore the impact of organizational safety management, superiors’ safety leadership, and employees’ safety attitude on safety performance. Therefore, safety performance is the ultimate mechanism to show whether safety maintenance is effective in an organization, and it is also a measure of how much importance an organization places on individual situations when facing safety and health issues as a whole (Majid et al., 2022). Taiwan’s military officers and soldiers should have safety performance in order to be called a qualified and quality military specification warfare force, because the achievement of safety performance will fully demonstrate that they are not afraid to face the threat of various kinds of warfare forces, and that there is a guarantee for both territorial and civilian safety considerations.

### 2.5. Relationship between sense-making theory and variables

The sense-making theory is of great importance to the organization sphere (Weick et al., 2005), which is widely utilized by scholars. The sense-making theory mainly explores the situation that people detect and extract specific clues from the environment, and interpret the clues based on their beliefs, mentalities, habits, conventions, and insider information when faced with a turbulent and complex environment and uncertainties. The sense-making of people when looking back to past events is a continuous process of action, selection, and interpretation, which involves cognition, emotion, and action; it is also a process of sharing cognition and understanding diverse views and interests (Jensen et al., 2009). People realize “only when I understand what I have said do I know what I was thinking” when constructing sense. People seek and maintain positive self-awareness and emotional state, affirming their value to the change in organizational environments (Dervin, 1998). Moreover, people are eager to feel that they are capable, making an effective response to the changes and reforms in an organizational environment. Faced with environmental changes, people strive to maintain the consistency between themselves and the environment, which means that they need to think about how to accept new values and beliefs brought by environmental changes while retaining their original ones so that the original ones will continue to have the value of existence (Savolainen, 1993). Sense-making often occurs when people try to maintain a consistent and positive self-concept. If people feel dissatisfied with current situations or their cognition is disturbed by the events around them, they will ask themselves the hints of the events for “who I am.” When people are thinking about what event is happening, they are making sense of the situations to them, and rid their dissatisfaction with the current situation by responding to the environment, such as adjustment, problem solving, and learning (Dervin, 1999). “Experience” means that when people look back on the past, the event that is happening at that time point, no matter what it is, exerts an impact on how they make sense of the past event. The environment can constrain people’s action. On the other hand, it provides people with opportunities. Moreover, people can create an environment themselves; when they do it, they are also getting to know the environment that they are in (Klein et al., 2006). Sense-making is not only an activity for an individual, but also a social one. When people engage in sense-making in the social context, their social resources, including reference groups, norms, rules, standards, events, ways of communication, and interaction with others in an organizational environment, affect their perception and interpretation of the environment (Thompson and Stapleton, 2009), and their information behaviors. Among these resources, interpersonal interaction is an easy way to obtain information, which is often useful to individuals (Stein, 2004). Consequently, social resources are often used by actors to construct the sense of an environment. The work within an organization is dynamic, so sense-making has no definite starting point; it is always an ongoing activity (Griffith, 1999). People extract certain clues from the ongoing activity for sense-making; if the activity is interrupted, people will experience emotional reactions, which affects their sense-making process (Keller, 2003). Emotions consist of positive and negative ones. Positive ones, encouragement and relief for instance, motivate people to find answers to questions, which differ because of their differences in socialization. By contrast, negative ones, such as confusion, worry, and uncertainty, make people take actions to dissolve them; people are often experiencing a negative emotion when making sense of an event. In brief, emotion, positive or negative, affects people’s perception and behaviors (Baran and Scott, 2010). The environment can affect the clues that people read, and how they explain the extracted clues. Moreover, their beliefs and values also affect clue filtering. The process of extracting clues includes searching, scanning, and noticing, while noticing refers to the activities of filtering, classifying and comparing clues. The sense-making theory does not specify clearly the clues in the environment, but it points out that organizational members will pay attention to the information, norms and interpersonal interactions within the organization, which indicates that clues exist in the environment. Viewed from the information sphere, environmental information is clues (Proulx and Inzlicht, 2012). If people combine the clues from the event that happens right now with the similar ones of a past event and interpret the clues one more time, they make sense of the past event again. People tend to combine current and previous clues to form present feelings based on the ones that they had; people expect to read the meaning of an event during sense-making. People understand subjectively the objects, remarks, and actions that they see or hear, which helps them understand the world. Consequently, people focus on their own real feelings when engaging in constructing sense (Davidson, 2010). Safety leadership, a kind of influence, enables organizational members to know the importance of safety. If safety is highly valued in an organization, one often gains risk perception, which conforms to the sense-making process. If this process become an enterprise system or part of organizational culture, it is showing the possibility of safety culture cultivation. Under the impact of safety leadership, organizational members will pay more attention to safety when carrying out tasks to achieve the performance; they will not sacrifice safety because of excessive focus on efficiency. Therefore, the study proposes the following hypothesis:

*H1*: Safety leadership exerts a significant positive impact on safety performance.

Safety leadership can be also interpreted as an opportunity for organizational change, which will make organizational members better understand the importance of safety because of the influence from leaders; they will be fully aware that risks may exist in every detail. In other words, the stronger the safety leadership, the clearer the risk perception of organizational members. Based on these, this study proposes the following hypothesis:

*H2*: Safety leadership has a significant positive influence on risk perception.

Meanwhile, organizational members will ponder over whether they are able to make a contribution to organizations (i.e., personal value) when the overall environment is experiencing changes, in other words, how to respond to the changing environment correctly. If the influence of safety leadership can achieve positive effects, one will take into consideration both safety and task completion during the implementation process. Consequently, organizational members with a deeper sense of risk perception will be more likely to think about how to finish each procedure safely, for which they will accomplish tasks successfully and effectively to achieve safety performance. That is to say, those with stronger risk perception tend to achieve better safety performance. Thus, this research puts forward the following hypothesis:

*H3*: Risk perception has a significant positive impact on safety performance.

The above statements have revealed that safety leadership provides an organization with a new signal that can be regarded as a sense-making process, which enables organizational members to know the importance and necessity of safety. With risk perceptions in mind, individuals will pay more attention to the details of their life, thus naturally improving safety performance. That is to say, the effect of safety leadership is produced based on the risk perceptions of each organizational member, which promotes the safety performance of each task. In other words, risk perception acts as a bridge between safety leadership and safety performance. Thus, this study proposes a hypothesis in this regard as follows:

*H4*: Risk perception mediates the relationship between safety leadership and safety performance.

From the sense-making theory, it can be known that the whole organization goes through an adaptive process to accept new meanings. If this process permeates the whole organization, it can grow into an organizational culture. In other words, if safety is seen as sense-making, the cultivated organizational culture can be involved in safety culture. If safety awareness permeates every corner of the organization or each procedure in an imperceptible manner, the organization will be able to lower its cost of safety monitoring, for all members within this organization will display safety behaviors without repeated communication or discipline from their superiors or authorized agents. Therefore, an organization with strong safety culture can enable safety leadership to place a greater impact on safety performance, which means that safety culture can strengthen the effect of safety leadership. Thus, this research proposes the following hypothesis:

*H5*: Safety culture mediates the relationship between safety leadership and safety performance.

**Figure 1:**
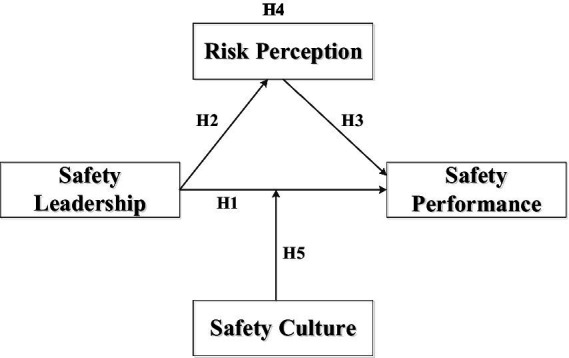
Research framework.

Based on literature review and deduction, this research discussed the relationship between variables and proposed five research hypotheses to later verification. This study illustrates the relationships between independent and dependent variables by presenting its research framework, as shown in Figure 1.

## 3. Research method

This research developed its research objective based on the research background and motivation after it confirmed the research topic. Having understood the research question and purpose, it started collecting relevant literature. Based on the literature review, this study confirmed its research scope. The research tools, methods, and participants are described in detail in the following sections.

### 3.1. Research participants and sampling method

This study recruited Taiwan military volunteer soldiers as its research participants. Taiwan military organizations spread across the country. To obtain representative samples, this study managed to get the phone numbers of army recruitment centers, asking them to help distribute questionnaires. In March 2020, 1,000 copies were mailed to the centers. In April 2020, 357 questionnaires were collected, while another 398 ones were gathered in May 2020. A total of 755 copies out of 1,000 were collected, of which 30 were deemed invalid after manual screening for missing responses and the same option for almost each question. Thus, 725 out of 755 questionnaires were valid, an effective recovery rate of 72.5%. Bentler and Chou (1987) proposed that the number of samples should be at least five times the estimated parameter while the estimated parameter is approximately twice the questionnaire items. The questionnaire for this study had 48 items, so 480 valid copies would suffice. This research obtained 725 valid ones, which meets the requirement.

### 3.2. Research instruments

This research compiled the questionnaire with question items from the scales that have been confirmed to have good validity and reliability. The items for safety leadership came from the research of Lu and Yang (2010); 12 items were selected as observed variables. When measuring risk perception, this research selected 12 items as observed variables from the research by Basha and Maiti (2013). It also selected 12 items from the scale developed by Herbert and Sarah (2013) to measure safety culture. It used 12 items to measure safety performance by referring to the research by Hadjimanolis and Boustras (2013). The scales all adopted five-point Likert scoring system with 1 being “strongly disagree,” while 5 being “strongly agree.” The higher participants score in a question, the higher the extent to which they perceive it mentally.

All items of the questionnaire measured their inner feelings with self-reported subjective cognition. The variables explored in this research cannot be measured with objective indicators. That is why participants were asked to answer the questions about these four variables to obtain relevant information. To avoid common method variance, question items for the same variable were dispersed throughout the questionnaire (Hinkin, 1998).

## 4. Result analysis and discussion

### 4.1. Descriptive statistics

Out of 725 valid samples, 584 were filled out by male respondents (accounting for 80.6%), while 141 were filled out by female (19.4%). The majority of them (a total of 279 respondents) were aged between 31 and 40 while 256 respondents, i.e., 35.4%, were based in north Taiwan. The majority of them (269 participants) were members of a land force, which accounted for 37.1%. Their demographic data are shown in Table 1.

**Table 1:** The demographic data of valid samples.

Variable	Percentage (Number)			
Gender	Male		Female	
	80.6% (584 respondents)		19.4% (141 respondents)	
Age	No more than 30	31–40	41–50	More than 50
	39.1%	38.5%	26.6%	4.2%
Region	North Taiwan	Central Taiwan	South Taiwan	East Taiwan
	35.4%	29.2%	34.7%	0.7%
Force	Land force	Naval force	Air force	Military police
	37.1%	28.7%	18.2%	16.0%

*n* = 725.

This section approaches the mean, standard deviation (SD), and reliability of each item. Each dimension and item of the questionnaire had a medium or high score, indicating that the participants identified the items to a quite high extent. The reliability for each dimension exceeded 0.7. Thus, the research participants all agreed to the items to a moderate extent or above. In addition, the questionnaire has good internal consistency (Kuijpers et al., 2013), as shown in Table 2.

**Table 2:** The mean, SD, and reliability for each question.

	Safety leadership	Mean	SD	Reliability
Visibility	The managers of the company that I am working for often go to factories for safety observation and inspection.	3.870	0.965	0.932
	The managers of the company actively participate in safety and health affairs.	3.840	0.981	
	The managers of the company pay much attention to safety and health and show their determination to implement safety policies and rules.	3.880	1.043	
Superior-subordinate relationship	The managers of the company can give full authorization, trusting and supporting their subordinates.	3.780	0.899	0.866
	The managers of the company discuss safety and health related issues with my direct superior.	3.840	0.979	
	The managers of the company participate in safety committee meetings, in which they listen to employees and take their advice.	3.600	0.939	
	The managers of the company encourage subordinates to participate in the discussion of safety and health issues, motivating the team to improve safety performance in a concerted effort.	3.840	1.029	0.866
Team input	The work team of the company know clearly about their safety and health responsibility and achieve the goal for safety performance.	4.110	0.830	
	The managers of the company encourage subordinates or work teams to participate in the compilation of safety and health strategies, planning, rules and standards.	3.840	0.876	
	The subordinates and supervisors of the company support the safety and health behaviors of the managers.	4.110	0.794	0.885
Proactive management	The managers of the company can spot the safety and health problems, stop unsafe behaviors immediately, and manage to improve workplace safety.	3.920	0.805	
	The managers of the company encourage subordinates to report workplace accidents rather than concealing them.	3.930	0.908	
	Risk perception	Mean	SD	Reliability
Safety awareness	My working partner knows clearly about how to respond to emergencies.	3.800	1.094	0.901
	My working partner encourages others to observe the safety and health rules.	3.890	1.010	
	My working partners pay much attention to the workplace safety.	4.100	0.968	
	I know clearly how to prevent the harm caused by machinery operation when running it.	4.220	0.774	0.865
Machinery	I know clearly the location of emergency stop switches on the machinery I operate.	4.170	0.886	
	I know the importance of daily equipment maintenance.	4.350	0.680	
	I conduct safety check before working.	4.100	0.833	0.936
Safety protection	I am clear about the way personal protective equipment is worn or used.	4.010	0.928	
	My working partner and I have suitable personal protective equipment.	3.740	1.172	
	I am clear about the location of hazardous substances.	3.930	1.061	0.805
Risk assessment	I am clear about how to react to accidents like explosion and fire.	3.900	0.925	
	I am clear about where fire extinguishers are put and how to operate them.	4.050	0.844	
	Safety culture	Mean	SD	Reliability
Management commitment to safety	The managers of the company pay attention to the health, safety, and welfare of the subordinates.	3.850	0.975	0.943
	I can apply the safety beliefs that the company often communicates to daily life.	3.990	0.801	
	The company I am working for organize emergency drills.	3.870	0.993	
	I follow the standard operating procedures that the company has set when working.	4.070	0.847	
Safety knowledge	The health and safety training enables me to acquire more safety knowledge.	4.140	0.812	0.909
	The health and safety training enables me to realize the hazards in my work.	4.170	0.751	
	I show support to the safety plans that my colleagues put forward.	4.260	0.654	
	I check regularly the equipment that I operate to ensure safety.	4.010	0.925	
	I do not drink during working hours or lunch hours.	4.430	0.928	0.704
	I observe traffic rules on my way to and from work.	4.250	0.885	
Response to emergencies	The division responsible for safety and health of the company formulates emergency plans.	4.220	0.814	
	Safety beyond working hours is covered by the safety policies of the company.	3.990	1.083	
	Safety performance	Mean	SD	Reliability
Safety unit	The division responsible for safety and health of the company announces safety policies in the form of text and clearly delegates safety responsibilities to each level.	3.980	0.909	0.918
	The division responsible for safety and health of the company responds positively to the occupational safety and health issues.	3.900	0.921	
Safety management	My work team often analyzes workplace safety and improves it.	3.960	0.957	0.944
	The division responsible for safety and health of the company proposes to refer to the physical check-up report of potential candidates when recruiting employees.	3.990	1.003	
Safety measures	The division responsible for safety and health of the company puts up signs and reminders in the workplace that requires protective equipment.	4.020	0.894	0.894
	The division responsible for safety and health of the company is able to purchase sufficient personal protective equipment for operators.	3.930	0.908	
Safety training	The division responsible for safety and health of the company offers general occupational safety and health training to new hires.	3.980	1.054	0.946
	The division responsible for safety and health of the company offers training in how to use newly purchased personal protective equipment.	3.840	1.142	
Safety equipment	The workplace of the company has adequate day lighting, artificial lighting and ventilation.	4.020	0.891	0.786
	The company often keeps the workplace clean and tidy.	4.070	0.923	
Incident investigation	The accident reports to the company are discussed and reviewed by the incident investigation committee or the occupational hazard assessment unit.	3.960	0.924	
	The division responsible for safety and health of the company keeps the record of accidents which will serve as examples for later employee training.	3.990	0.909	

### 4.2. Confirmatory factor analysis

This study refers to the scales of previous researches, which have been utilized in relevant researches multiple times. In this aspect, the content validity of the scales is guaranteed, for each question was compiled based on a solid theoretical foundation. To confirm that each scale has good reliability and validity, confirmatory factor analysis (CFA) was adopted for estimation and their convergent validity was reviewed by composite reliability (CR) and average variance extracted (AVE).

The CFA results showed that the factor loadings (FL) for observed variables all exceeded 0.5 while the indicator reliability (IR) all approximated 0.5, indicating that the CFA model had a good fit. The CR and AVE results showed that safety leadership had a CR of 0.923 and an AVE of 0.751, risk perception had a CR of 0.878 and an AVE of 0.645, safety culture had a CR of 0.910 and an AVE of 0.772, and safety performance had a CR of 0.965 and an AVE of 0.822. The convergent validity (CV) of all variables passed the standard (Fornell and Larcker, 1981), as shown in Table 3.

**Table 3:** CFA and convergent validity.

Dimension	Construct	Parameter estimation				FL	IR	CR	CV
		UnStd	S.E.	*t*-value	*P*	Std	SMC	CR	AVE
Safety leadership	Visibility	1.000				0.872	0.760	0.923	0.751
	Superior-subordinate relationship	0.770	0.024	31.756	***	0.834	0.696		
	Team input	0.766	0.023	33.915	***	0.860	0.740		
	Proactive management	0.731	0.019	37.654	***	0.899	0.808		
Risk perception	Safety awareness	1.000				0.859	0.738	0.878	0.645
	Machinery	0.730	0.028	26.303	***	0.801	0.642		
	Safety protection	1.062	0.035	30.566	***	0.887	0.787		
	Risk assessment	0.696	0.036	19.091	***	0.643	0.413		
Safety culture	Management commitment to safety	1.000				0.867	0.752	0.910	0.772
	Safety knowledge	0.907	0.027	33.580	***	0.907	0.823		
	Response to emergencies	0.840	0.027	30.906	***	0.861	0.741		
Safety performance	Safety unit	1.000				0.952	0.906	0.965	0.822
	Safety management	1.067	0.022	49.137	***	0.919	0.845		
	Safety measures	0.951	0.020	47.469	***	0.911	0.830		
	Safety training	1.178	0.026	45.586	***	0.902	0.814		
	Safety equipment	0.890	0.021	42.347	***	0.882	0.778		
	Incident investigation	0.950	0.023	40.777	***	0.872	0.760		

*p<0.05, **p<0.01, ***p<0.001.

In addition to convergent validity, the correlation coefficients of the latent variables were compared with the square root of their AVEs. If the square roots of the AVEs of two variables are greater than their correlation coefficient, the variables have preferable discriminant validity. In this study, all square roots of the AVEs were larger than corresponding correlation coefficients, so each variable has discriminant validity (Farrell, 2010), as shown in Table 4.

**Table 4:** The correlation coefficients and discriminant validity for each variable.

	CR	AVE	Safety culture	Safety leadership	Risk perception	Safety performance
Safety culture	0.910	(0.772)	(0.879)			
Safety leadership	0.923	0.751	0.650	(0.867)		
Risk perception	0.878	0.645	0.227	0.580	(0.803)	
Safety performance	0.965	0.822	0.213	0.544	0.190	(0.907)

The diagonal value is the square root of the AVE for each construct.

### 4.3. Path analysis of latent variables

**Figure 2:**
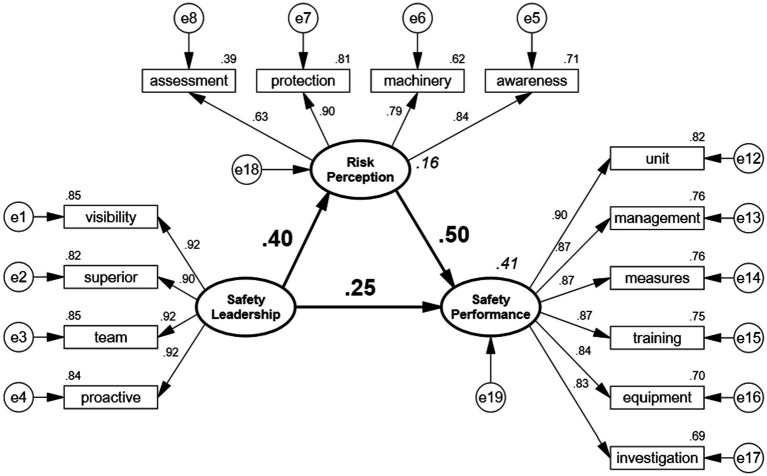
Latent variable model and path analysis of standardized coefficients.

This study adopted the maximum likelihood method of the structural equation modeling to estimate path coefficients and effect sizes. This way, most hypotheses were verified and the model fitness indexes all reached the standard. For the structural model overall, the Chi-square value was 89.327, the degree of freedom was 77. Thus, the ratio of Chi-square to the degree of freedom (χ^2^/df), i.e., normed Chi-square, was 1.160, which is less than 5, indicating a level of significance. Moreover, GFI equals to 0.994, AGFI 0.991, CFI 0.994, NNFI 0.940, and IFI 0.930, which all exceed 0.90. In addition, RMSEA equals to 0.015, which is less than 0.08. To conclude, the values of all indicators fell within a reasonable range (Jackson et al., 2009). Consequently, the model has a good fit, as shown in Figure 2.

The structural equation modeling provides multiple indicators that test whether a model has a good fit, of which CFA, NNFI, RMSEA, and SRMR are the most important ones. The indicators for this research all reach the standards, as shown in Table 5.

**Table 5:** Model fitness indexes.

Fitness index	Ideal value	Model fit level
Bollen-Stine *χ*^2^	Lower is better	89.327
DF (Degree of Freedom)	Higher is better	77
Normed Chi-square (*χ*^2^/DF)	1 < *χ*^2^/DF < 3	1.160
GFI	>0.9	0.994
AGFI	>0.9	0.991
RMSEA	<0.08	0.015
SRMR	<0.08	0.079
TLI (NNFI)	>0.9	0.940
CFI	>0.9	0.994
IFI	>0.9	0.930
Hoelter’s N (CN)	>200	626.264

This research mainly explores the relationship among safety leadership, risk perception, safety culture, and safety performance with military volunteer soldiers as its research participants. Having confirmed that the reliability and validity all reached the standards, the path analysis of latent variables was employed to estimate standardized coefficients (SC) (Kline, 2015), finding that all path coefficients reached the level of significance. Specifically, the standardized coefficient of safety leadership to safety performance was 0.248 (*p* < 0.01) and the confidence interval (CI) did not include 0, indicating that safety leadership has a positive impact on risk perceptions, so H1 is validated. Moreover, the standardized coefficient of safety leadership to risk perceptions was 0.399 (p < 0.01) and the CI did not include 0, indicating that safety leadership exerts a positive effect on risk perception, so H2 is supported. As for H3, the standardized coefficient of risk perception to safety performance was 0.497 (p < 0.01) and the CI did not include 0, indicating that risk perception places a positive effect on safety performance, so the hypothesis is validated. In brief, the first three hypotheses that this research proposed have all been validated, as shown in Table 6.

**Table 6:** Standardized path analysis of variables and significance.

Parameter	Estimate	Bias-corrected		Percentile		Sig.
		Lower	Upper	Lower	Upper	*p*
Risk perception ← Safety leadership	0.339**	0.369	0.428	0.371	0.430	0.007
Safety performance ← Safety leadership	0.248**	0.234	0.264	0.234	0.264	0.004
Safety performance ← Risk perception	0.497**	0.460	0.541	0.456	0.539	0.002

^*^p < 0.05, ^**^p < 0.01, ^***^p < 0.001.

### 4.4. Analysis of mediation effects

To further explore the mediation effect of risk perception on the relationship between safety leadership and safety performance, bootstrapping was employed to compute standard errors of total and indirect effects and the CI of all effects; if the CI of an effect does not include 0, then the effect is significant (Henseler et al., 2015).

If the CI of the total effect does not include 0, the total effect is significant, while the indirect effect also is significant if its CI does not include 0, either (Hooper et al., 2008). The research results showed that the indirect effect of safety leadership → risk perception → safety performance was 0.198 and the CI of mediation effects did not include 0, suggesting that the mediation effect is significant. Thus, H4 is supported.

The total effect was 0.447, of which the direct effect (safety leadership → safety performance) was 0.248, accounting for 55.48% of the total effect and the indirect effect (safety leadership → risk perception → safety performance) was 0.198 (i.e., 44.30%). Therefore, this model has great explanatory power for the relationship between safety leadership and safety performance, as shown in Table 7.

**Table 7:** Verification of mediation path model and effect analysis.

Effects of the model	Point estimate	Product of coefficient		Bias-corrected		Percentile		Sig.
		SE	*Z*	Lower	Upper	Lower	Upper	*p*
Direct effect (leadership → performance)	0.248	0.008	31.000	0.234	0.264	0.242	0.276	0.011
Indirect effect (leadership → risk → performance)	0.198	0.006	33.000	0.187	0.211	0.186	0.223	0.006
Total effect (direct effect + indirect effect)	0.447	0.014	31.929	0.420	0.475	0.420	0.456	0.015

Based on the statistical analysis method by Aiken and West (1991), the predictive variable X and the moderator variable M were both standardized and multiplied. If the interaction variable (X multiplied by M) had a significant predictive effect on the dependent variable Y, it indicated the existence of the moderation effect. After that, the interaction diagram was drawn to review the interactions.

To verify whether safety culture mediates the relationship between safety leadership and safety performance, this study adopted multiple hierarchical regression analysis (MHRA) for testing. Firstly, the variables of safety leadership and safety culture were both transformed into Z-scores. Then, the two Z-scores were multiplied, and the corresponding result was substituted into the regression equation for verification. The results are shown in Table 8.

**Table 8:** Mediation effect of safety culture on the relationship between safety leadership and safety performance.

Model	Independent variable	Unstandardized coefficients	SD	Standardized coefficients	*t*	Sig.
		Estimated B		Beta distribution		*p*
1	(Constant)	3.971	0.017		227.223	0.000
	Safety leadership	0.713	0.017	0.835***	40.774	0.000
2	(Constant)	3.971	0.012		343.823	0.000
	Safety leadership	0.056	0.024	0.066*	2.295	0.022
	Safety culture	0.746	0.024	0.873***	30.552	0.000
3	(Constant)	4.062	0.015		270.666	0.000
	Safety leadership	0.074	0.023	0.086**	3.166	0.002
	Safety culture	0.701	0.024	0.820***	29.513	0.000
	Leadership × Culture	−0.103	0.012	−0.120^***^	−8.880	0.000

Dependent variable: safety performance. ^*^p < 0.05, ^**^p < 0.01, ^***^p < 0.001.

**Figure 3:**
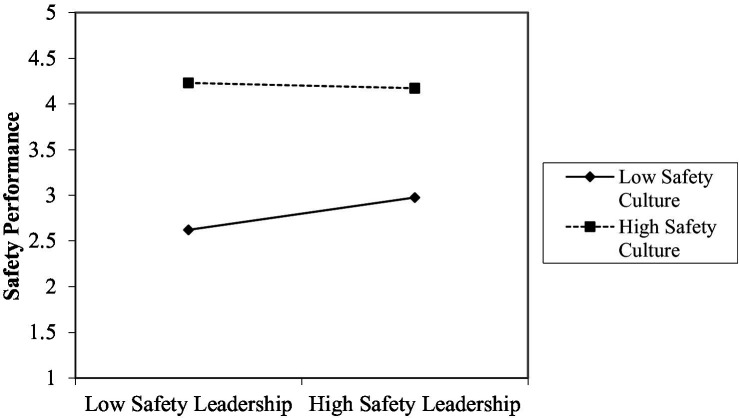
The mediation effects of safety culture at different levels.

The MHRA was conducted, revealing that the interaction item of safety leadership and safety culture produces a significant impact on safety performance, which validates H5, as shown in Table 8. To further understand the mediation effect, a diagram was drawn with an Excel spreadsheet, finding that safety leadership does not have a significant impact on safety performance when the safety culture is at a relatively high level. However, when the safety culture is at a relatively low level, safety leadership exerts a significant positive impact on safety performance, as shown in Figure 3.

## 5. Conclusion and recommendation

Safety leadership and risk perception have been found to exert an impact on safety performance. Risks are people’s judgments about the chance that accidents would happen. Their judgments are susceptible to subjective thoughts, suggesting that leaders’ communication and implementation of safety policies would greatly improve workplace safety. In addition, risk perception has a partial mediation effect on the relationship between safety leadership and safety performance, suggesting that safety leadership can improve safety performance through enhancing the risk perception of organizational members. This finding reveals the importance of risk perception enhancement to safety performance. As for military volunteer soldiers, they will exhibit expected safety behaviors like safety check, regular equipment maintenance, and following standard operational procedures when performing tasks if they have great awareness and clear perception of workplace safety. As well, the impact of safety leadership is also very important; managers’ planning, monitoring, and examination of safety and health at workplaces will enable organizational members to achieve better safety performance.

Safety culture does mediate the relationship between safety leadership and safety performance. However, it is noteworthy that safety leadership does not have a significant impact on safety performance if safety culture is at a high level, whereas it exerts a significant positive influence on safety performance if the safety culture is at a low level. The research result is not consistent with the hypothesis, but it reveals that organizational members can be unconsciously influenced by safety culture. It is possible that all military volunteer soldiers internalize safety awareness and need no monitoring from managers when safety culture has been fostered within a military organization, thus keeping safety performance at a high level. In other words, when an organization has not cultivated safety culture, the superiors of an army need to show great safety leadership, imparting the importance of safety to their subordinates and providing safety training. Only in this way will an army improve safety performance under the influence of safety leadership. The results of the present study can provide reference to (1) military volunteer soldiers when they check their own safety awareness, and (2) military organizations if they introduce safety leadership to their management based on safety culture. Safety leadership can be incorporated into national defense policies, which facilitates the implementation of safety rules and practices. This way, military volunteer soldiers will fulfill their duties and complete tasks in a safe manner and grow into a high-quality army.

In addition to traditional military threats, the concept of national security defense has changed significantly in the 21st century, with the proportion of non-military threats increasing year by year. Terrorism, financial crisis, energy competition, extreme climate, natural disasters, food shortages, and new infectious diseases have caused more casualties than war and have a greater impact on national security than ever before. The impact not only changes the type of warfare, but also extends the scope of national security to political, economic, social, and psychological aspects, without distinguishing between peacetime and wartime, and without distinguishing between frontline and rear line. Therefore, the duty to protect national security is no longer the sole responsibility of military personnel, but the common responsibility of all citizens. It is the common responsibility of all citizens. It is urgent to make the national defense awareness deeply rooted in people’s hearts and minds, and to implement it in daily life.

There is no holiday in Taiwan’s national defense and war preparation, and it is important to have a long-term strategic goal, and at the same time, there must be a certain period of time and related supporting actions. However, it cannot be denied that due to the long training without war and the influence of the surreal changes in cross-strait relations, it is indeed necessary to strengthen the officers’ and soldiers’ awareness of the enemy, enhance their sense of worry, and realize the truth that only “war preparation can stop war,” so as to implement “training for war” and strive for the original task of war training. “In order to build a solid national defense brigade, officers and soldiers of the national army should continue to improve their academic skills in the spirit of perfection, and to learn the knowledge and skills of modern technology management, with the aim of building a quality brigade with “less quantity, better quality, and stronger combat capability” to provide a more solid guarantee for national security and social stability. At this stage, our national defense policy is based on the basic concept of “preventing war, maintaining stability in Taiwan and the sea, and safeguarding homeland security.” The officers and soldiers of the national army should always uphold the attitude of “preparing for war without seeking war, stopping war without fearing war,” and “not provoking or evading,” and focus on their work of war training with the spirit of “improving, seeking practicality, being strict, and dealing with difficulties” in a pragmatic and responsible manner, and work hard to build the army and prepare for war. Although we have no intention to engage in an arms race with the Chinese Communist Party, in order to ensure the survival and development of our country, we still need to build a self-reliant national defense force and implement the construction of a modern national defense in order to enhance the overall national defense capability.

## Data availability statement

The raw data supporting the conclusions of this article will be made available by the authors, without undue reservation.

## Author contributions

S-YW: conceptualization, writing original draft, and review and editing. Y-KK: data collection and empirical estimations. All authors contributed to the article and approved the submitted version.

## Conflict of interest

The authors declare that the research was conducted in the absence of any commercial or financial relationships that could be construed as a potential conflict of interest.

## Publisher’s note

All claims expressed in this article are solely those of the authors and do not necessarily represent those of their affiliated organizations, or those of the publisher, the editors and the reviewers. Any product that may be evaluated in this article, or claim that may be made by its manufacturer, is not guaranteed or endorsed by the publisher.
